# Abstract of the Proceedings of the Buffalo Medical Association

**Published:** 1870-09

**Authors:** Wm. C. Phelps

**Affiliations:** Secretary


ART. II.—*Abstract of the Proceedings of the Buffalo Medical Association.*

The President and Vice-President being absent, Dr. P. H. STRONG was invited to take the chair.

Dr. MINER had invited a patient of his to be present, in order to show a remarkable recovery after compound fracture of the lower ends of the tibia and fibula. Twelve weeks since, the patient was caught in a boat line, and the two bones separated at their lower epiphyses, and driven through the soft parts upon the inner side of the leg, making great laceration and contusion of the muscles, and denuding the protruded portion of the bones of periosteum. The patient was directed to him for the purpose of amputation, as the injury was such that no expectation of saving the leg was entertained. Supposing that the circulation might not be greatly interfered with by the injury, he concluded to replace the bones, and wait for indications. About two weeks after injury, no severe constitutional symptoms having been developed, and finding that portion of the bones denuded of periosteum, in a bare and carious condition, he made exsection of two inches in length of the bones, dividing them with chain saw in healthy portion. The leg was laid in natural position, and carbolic acid lotions constantly applied. No unpleasant symptoms occurred, and the recovery had been as rapid as in any case of compound fracture; indeed the leg is now as strong and useful as is common at the same period after simple fracture. He said the most remarkable feature of the case is the small amount of shortening after exsection of two inches of bone. The best measures show shortening of only one inch, and the pelvic compensation is so complete, that the patient scarcely shows the loss, in his gait; will not show it at all after a little time. This must be due to deposite of new osseous material, compensating for one half of the portion removed. He thought the separation of bone at the epiphysis would more likely be followed by such compensation than in fracture proper. The young man was eighteen years old, and the bone does not become completely ossified until the twenty-first or twenty-second year, hence the diastasis instead of fracture.

The case appeared interesting, mainly, since 1st—Such injuries were formerly subject to amputation, and but little trial made with a view to save the leg. 2nd—The amount of shortening being but one half of the exsected bone. 3rd—The rapid recovery, and present good use of the leg; and 4th—The slight amount of deformity, and perfect motion preserved in the ankle joint.

Dr. STRONG spoke of the conservatism of the present practice of surgery, as compared with the past, and believed great progress had been made in this respect. A mere glance at the leg was sufficient to show an unusually satisfactory result. He believed that formerly such injuries were almost uniformly doomed to amputation, and that even now, many surgeons would advise amputation. Such cases would go far to establish the rule, never to amputate for injury to bone when the integrity of the circulation was perfect.

Dr. PHELPS related the following case:—On the morning of the 1st of June last, I was called to visit Joseph Lynch, aged eighteen months, who had the preceding evening fallen from a window, in the third story of the Union Block, on Commercial Street, striking the left side of his head on the stone flagging which composed the sidewalk. He was at once carried in an insensible condition to the residence of his parents, and two physicians called. They were of opinion that the child could live but a short time, and not thinking it advisable to use any means for his restoration, went away. During the night there was no perceptible change in the condition, but as he was still alive in the morning, the father called me to see him.

I found the left parietal bone fractured, but not depressed, and there were none of the usual symptoms of compression of the brain. There was a large swelling of the integument at the seat of fracture. The condition of the patient was that of great shock. Evaporating lotions, and other measures to limit inflammation, were directed, and the patient was soon convalescent, but his sight was totally destroyed. There was nothing abnormal in the external appearance of the eye, and to discover the cause of blindness, Dr. Abbott was requested to make an ophthalmoscopic examination of them. Nothing abnormal was discovered by this examination, all the parts composing the interior of the eye being entirely healthy. The seat of the injury, therefore, which caused the loss of sight, is posterior to the eye—probably in the brain—being due to the great concussion which it sustained by the fall. Aside from the loss of sight, the child is now in excellent health.

Dr. MINER exhibited a tumor, which, assisted by Dr. Dayton, he had removed from the orbit the day previous. It was found in a healthy man of about thirty-five years of age. It was not attended with pain; was of two years standing, and before being placed in alcohol, was about the size of the globe of the eye. It was found to occupy the place of the globe, and to have displaced the eye upwards, so that the eye was entirely crowded out of its normal position. Vision remained enough to be able to determine light, and to show that the integrity of the nerve and retina was not wholly destroyed. Ophthalmoscopic observation was not made, as the organ was not in position to allow it, and the lower portion of the cornea, which might have been reached, had become opaque, from not being covered or washed by the lids.

The removal of the tumor was accomplished by dividing the conjunctiva and sub-conjunctival tissue of the lower lid, down to the tumor, extending the incision through the outer canthus. The growth was then carefully separated from the parts with the handle of the scalpel and finger, and removed but with little dissection of fibrous attachments, with scissors The hemorrhage was profuse at first, but soon ceased, and was not very troublesome. The question of removing the globe, in order to remedy deformity, and insure the safety of the other eye, was considered, but by unanimous consent of all present, it was left, with the view that if it did not resume its normal position, or should in other respects prove unsafe or objectionable, it could be as safely removed at a subsequent operation. The lids were now drawn as fully over the globe as possible, and retained by adhesive plaster and compress, and water dressings applied.

The tumor is remarkable in its appearance, and one might, by superficial examination, have a wrong impression of its nature. Its history and defined border indicated that it was not malignant; but in order to determine its true nature, he had asked Prof. Hadley to make microscopic examination of it, which, being very carefully conducted, showed it to be fibrous in character It might then be regarded as a specimen of fibrous tumor of the orbit, a form of disease described by all authors upon orbital tumors. He thought malignant growths, having their origin in, or first appearing from the orbit, the most common, as he had met with several cases. The fatty and fibrous growths were also occasionally met by surgeons, but it was by no means a common site for either of these growths.

The amount of vision which would be present, or that the globe would fully return to its normal place, could not yet be determined; a few days, however, would settle both these questions.*

Dr. Abbott remarked upon the rarity of such growths, and the various points of interest in the case, approving of the plan of leaving the eye to return to its normal position, or, if found necessary, to remove after its necessity was fully apparent.

Dr. Miner then presented an eye which he had enucleated for sympathetic ophthalmia. It was removed from a gentleman from

* The globe at the present time, ten days after the operation, has resumed its natural position. The exact amount of vision has not been noted, but he says " he can see objects distinctly," and that he could " make his way in the world with that eye alone." The cornea is now fully covered with the lids, and its transparency is being rapidly restored.

North East, Pa., who had received a blow two months before, immediately destroying vision. The globe had softened and sensibly atrophied, and been the seat of a low form of inflammation, in all its tissues. The healthy eye had become also the seat of a very low form of inflammation, which had not greatly impaired the motions of the iris, or perceptibly changed the structure of the retina.

The injured eye was hopelessly lost, and all effort was now to be directed to the protection of the sound one. Photophobia, lachrymation, slightly changed iris, and dimness of vision were symptoms too indicative of sympathetic inflammation to be neglected, and the importance of removing the injured eye was urged upon the patient. He said he had too frequently and too recently urged upon the members of the society the importance of early recognising and properly treating such cases, to make it proper to again present the subject in such detail as its importance would otherwise demand, he would, therefore, only show the condition of the enucleated eye, and suppose that "a word to the wise would be sufficient." Upon section of the globe (now hardened by having been placed in equal parts of alcohol and water for twenty hours), may be seen the changes which the injury produced in the organ. The vitreous body had changed into a thin straw colored serum, the globe was thus softened, and atrophied. The pupil was closed by inflammatory products, and there was a very extensive effusion of blood between the choroid and retina, separating these two membranes throughout nearly their entire extent. A moment's observation of the enucleated globe would show the propriety and absolute necessity of its removal to preserve unimpaired vision in the healthy eye.

Adjourned.

Wm. C. Phelps, *Secretary*.

